# Experimental infection of Egyptian rousette bats *(Rousettus aegyptiacus)* with Sosuga virus demonstrates potential transmission routes for a bat-borne human pathogenic paramyxovirus

**DOI:** 10.1371/journal.pntd.0008092

**Published:** 2020-03-02

**Authors:** Brian R. Amman, Amy J. Schuh, Tara K. Sealy, Jessica R. Spengler, Stephen R. Welch, Shannon G. M. Kirejczyk, César G. Albariño, Stuart T. Nichol, Jonathan S. Towner

**Affiliations:** 1 Viral Special Pathogens Branch, Centers for Disease Control and Prevention, Atlanta, Georgia, United States of America; 2 Emory University, Yerkes National Primate Research Center, Atlanta, Georgia, United States of America; 3 University of Georgia, College of Veterinary Medicine, Athens, Georgia, United States of America; Colorado State University, UNITED STATES

## Abstract

In August 2012, a wildlife biologist became severely ill after becoming infected with a novel paramyxovirus, termed Sosuga virus. In the weeks prior to illness, the patient worked with multiple species of bats in South Sudan and Uganda, including Egyptian rousette bats (ERBs: *Rousettus aegyptiacus*). A follow-up study of Ugandan bats found multiple wild-caught ERBs to test positive for SOSV in liver and spleen. To determine the competency of these bats to act as a natural reservoir host for SOSV capable of infecting humans, captive-bred ERBs were inoculated with a recombinant SOSV, representative of the patient’s virus sequence. The bats were inoculated subcutaneously, sampled daily (blood, urine, fecal, oral and rectal swabs) and serially euthanized at predetermined time points. All inoculated bats became infected with SOSV in multiple tissues and blood, urine, oral, rectal and fecal swabs tested positive for SOSV RNA. No evidence of overt morbidity or mortality were observed in infected ERBs, although histopathological examination showed subclinical disease in a subset of tissues. Importantly, SOSV was isolated from oral/rectal swabs, urine and feces, demonstrating shedding of infectious virus concomitant with systemic infection. All bats euthanized at 21 days post-inoculation (DPI) seroconverted to SOSV between 16 and 21 DPI. These results are consistent with ERBs being competent reservoir hosts for SOSV with spillover potential to humans.

## Author summary

Sosuga virus (SOSV) was first identified in August 2012 in a sample obtained from a biologist that had become severely ill after working with bats in Sudan and Uganda. Testing of bat tissues from Uganda revealed the presence of SOSV RNA in Egyptian rousette bats (ERB: *Rousettus aegyptiacus*). To more definitively determine if these bats could be reservoir hosts of SOSV with potential for transmitting the virus to humans, we subcutaneously inoculated 12 captive-bred ERBs with a recombinant SOSV and collected biological samples daily through 21 days post inoculation (DPI) to investigate the dynamics of virus infection and shedding. All bats became infected with SOSV and exhibited only mild cellular pathology following histopathological analyses. Multiple qRT-PCR and virus isolation positive tissues were collected as well as urine, oral, rectal, and fecal swabs, indicative of a systemic infection. At study completion, no animals had displayed any overt clinical signs of infection, virus was detected in multiple tissues, and all infected bats euthanized at the latest time point had seroconverted to SOSV. Collectively, our study demonstrates ERBs are a competent host and exhibit attributes consistent with being a natural reservoir and zoonotic source for SOSV.

## Introduction

Bats (order Chiroptera) have been associated with paramyxoviruses for over half a century. Parainfluenza virus was first isolated from a Leschenault’s Rousette bat (*Rousettus leschenaultii*) in 1966 [1] and Mapuera virus was isolated from salivary gland tissue of a little yellow-shouldered bat (*Sturnira lilium*) captured in Brazil in 1979 [2]. Since then, an increasing number of bat species have been identified as hosts of numerous paramyxoviruses [3]. Bats are second only to rodents in species diversity and account for nearly one quarter of known mammals on the planet, with representatives on every continent and landmass except Antarctica and some remote islands [4]. Bats from the chiropteran family Pteropodidae have been identified as natural reservoirs of two known and severe human pathogenic paramyxoviruses, Nipah virus (NiV) and Hendra virus (HeV) [5–8]. Experimental inoculations of pteropid bats with NiV produced subclinical infection, viral shedding in urine, and eventual seroconversion without overt signs of morbidity, consistent with expectations for a natural reservoir [9].

*Paramyxoviridae* is a large and diverse family of viruses. Currently, the International Committee on Taxonomy of Viruses recognizes 69 species of paramyxoviruses with much of the diversity spread between two subfamilies: *Avulavirinae* (20 species) and *Rubulavirinae* (17 species) [10]. Several pathogenic species of paramyxoviruses affect numerous animal taxa, including humans [3, 11–14]. Recently, a new human pathogenic paramyxovirus, Sosuga virus (SOSV), was identified when a wildlife biologist became severely, but non-fatally ill after performing field work with bats in South Sudan and Uganda in 2012 [11]. Testing of Ugandan bats from multiple bat species captured in the three-week period prior to the patient’s symptom onset, as well as archived bat tissues collected during previous outbreak investigations in Uganda, revealed that Egyptian rousette bats (ERBs: *Rousettus aegyptiacus*; family Pteropodidae) were the only bats to test positive for SOSV RNA[15].

After finding SOSV RNA in ERB tissues [15], this bat species was suspected to be the SOSV natural reservoir. However, since no routes of virus shedding were identified and no infectious SOSV was isolated from the wild-caught ERBs, its status as a natural reservoir remained unclear. Therefore, to more fully address these questions, captive bred ERBs were experimentally inoculated with a recombinant SOSV [16], identical to the human virus sequence, and then serially sacrificed to measure the kinetics of viral replication and shedding. The findings of this study are reported herein.

## Materials and methods

### Ethics statement

All experimental procedures described here were performed following methods previously reported [17] with slight modifications and were conducted with approval from the Centers for Disease Control and Prevention (CDC, Atlanta, GA, USA) Institutional Animal Care and Use Committee (protocol number 2874TOWBATC), and in strict accordance with the Guide for the Care and Use of Laboratory Animals (Committee for the Update of the Guide for the Care and Use of Laboratory Animals, National Research Council of the National Academies 2011).

### Animals and biosafety

The CDC is an Association for Assessment and Accreditation of Laboratory Animal Care International fully accredited research facility. No human patient-derived clinical materials were used in the completion of these studies. In compliance with US CDC policy, this study was reviewed and approved by the CDC Institutional Biosafety Committee.

Procedures conducted with infectious SOSV or with SOSV-infected bats were performed at the CDC under a minimum of biosafety level 3 (BSL-3) laboratory conditions, in accordance with all safety regulations. All investigators and animal handlers followed strict BSL-3 plus biosafety and infection control practices to prevent cross-contamination between experimentally infected and control bats.

All bats used in this study were captive-born bats from a SOSV-free breeding colony founded from wild-caught ERBs imported from Uganda. The experimental group consisted of 15 juvenile males 4–5 months of age, with an average weight of 112 g. The bats were housed in groups of no more than 6 as previously described [17].

### Virus stock

A recombinant SOSV generated by reverse genetics [16] and genetically identical to the virus RNA extracted from an acute-phase patient sample was used in this study. The virus was diluted in a solution of Dulbecco’s modified Eagle’s medium (DMEM) to a concentration of 1×10^4^ tissue culture infective dose 50 (TCID_50_)/250 μL.

### Experimental design

The bats (n = 15) were randomly divided into 2 groups consisting of virus-inoculated bats (n = 12) and mock-inoculated control bats (n = 3). Baseline blood samples, body weights, and temperatures were taken prior to inoculation. All bats were anesthetized with isoflurane prior to inoculation. Bats were inoculated via subcutaneous injection in the mid-ventral abdomen with either 250 μL of SOSV prepared in DMEM for a total dose of 1x10^4^ TCID_50_ per bat (n = 12), or 250 μL of sterile DMEM for control bats (n = 3). The rationale behind using a subcutaneous route of inoculation was to use a method of internal inoculation that ensures a uniform dose and that mimics a potential natural exposure in the wild, in this case, a bite or a scratch.

Rectal temperatures and body weights were recorded daily. Blood, oral and rectal swab samples were collected daily for subsequent RNA extraction and PCR analysis. Urine and fecal samples were collected opportunistically. Infected bats were euthanized under anesthesia via cardiac exsanguination and full post-mortem necropsies were performed on 3 infected bats on days 3, 6, 9, and 21 post-inoculation (DPI). The 3 negative control bats were euthanized and similarly processed at 21 DPI. Cardiac blood was taken upon euthanasia for downstream analysis. Complete tissue sets were taken and either stored in RNA extraction buffer for PCR analysis, or frozen and stored under liquid nitrogen vapor. All statistical analyses and graph construction were performed using GraphPad Prism 6 software (La Jolla, CA).

### Post-inoculation procedures

Body weights and temperatures of all bats were measured and recorded from 1 DPI through the end of the study. A temperature probe covered with a plastic sheath (MABIS Healthcare, Waukegan, IL, USA) was used to take the rectal temperature of each bat. The plastic sheath was then cut and placed into a deep-well plate with 500 μL of MagMax lysis buffer solution (Life Technologies, Grand Island, NY, USA) for PCR analysis. Two polyester-tipped applicators (Fisher Scientific, Grand Island, NY, USA) were used to swab the oral cavity of each bat. The swabs were immediately placed in either a deep-well plate with 500 μL of lysis buffer solution for PCR analysis, or in viral transport medium for virus isolation. Daily blood samples (≤60 μL) were taken via venipuncture from the cephalic vein on the propatagium, alternating left and right wings each day. Necropsies were performed on the aforementioned euthanasia days to collect the following tissues: skin, axillary lymph node, salivary gland, testis, large intestine (colon/rectum), small intestine, liver, spleen, kidney, heart, lung, eyes, brain, and bone marrow. Following tissue collection for PCR, all remaining tissues along with the bat carcasses were immersed in 10% neutral buffered formalin for histopathology. The tissues were fixed in formalin in the high containment laboratory for a minimum of 7 days, with the formalin being completely replaced at least 3 days prior to further processing.

### Sample testing and RNA extraction

All tissue, blood, and swab samples were analyzed by quantitative reverse transcriptase PCR (qRT-PCR) using reagents and procedures described previously [18], but modified to detect the SOSV N gene [15] using the following primers: PMX-2012 FOR3—GAT TCG AAC CGG GAA CAT AC; PMX-2012 REV3—TGG GAT ATC ATC TGC CAT TTC; PMX-2012 PRB3—FAM-TGC AAG AAC/ZEN/ ACC GCT GAC AGT AGG-IABkFQ. For tissue specimens, approximately 100 mg were placed in 2 mL grinding vials (OPS Diagnostics, Lebanon, NJ, USA) with 1 mL of MagMax lysis buffer concentrate and homogenized. Specimen sizes of some tissues (axillary lymph node and testis) were under 100 mg due to availability, but remained consistent within a tissue type. Bone marrow was flushed from the radius using 5 mL PBS, from which 100 μL of the marrow in MagMax lysis buffer concentrate was used for PCR. Total RNA extraction was achieved using the MagMax-96 Total RNA Isolation kit (Life Technologies) per manufacturer’s recommendations and the AM1830_DW protocol pre-loaded on the MagMax Express-96 Deep Well Magnetic Particle Processor. Each reaction used 125 μL of the tissue lysate with 75 μL of 100% isopropanol. To account for sample-to-sample variation, qRT-PCR results for tissues were normalized to results for 18s rRNA using a commercially available eukaryotic 18s rRNA primer/probe assay (Applied Biosystems, Grand Island, NY, USA) according to the manufacturer’s instructions. Gamma-irradiated Rift Valley fever virus (RVFV; 0.25 μL per sample) was added to the lysis buffer solutions as an internal extraction control for blood, swab, and urine samples. The RVFV qRT-PCR was performed as previously described [19], but using 58 °C for the annealing temperature.

Blood samples (20 μL; whole, non-heparinized) were added to 130 μL of MagMax lysis buffer solution (1:1 ratio of lysis buffer concentrate and 100% isopropanol). RNA was extracted using the MagMax-96 Viral RNA Isolation kit and the AM1836_DW_50v2 protocol pre-loaded on the MagMax Express-96 Deep Well Magnetic Particle Processor.

Polyester-tipped applicators used as oral swabs, plastic probe cover sheaths used as rectal swabs, and urine samples (50 μL) were placed into wells of a deep-well plate containing 500 μL of lysis buffer solution. Viral RNA was extracted using the 5× MagMax Pathogen RNA/DNA kit per manufacturer’s instructions, using 200 μL sample and 500 μL lysis binding solution, and the 4462359_DW_HV protocol pre-loaded on the MM-96 processor.

Standard curves for qRT-PCR results were generated by performing serial 10-fold dilutions of SOSV stock of known titer (TCID_50_/mL) in DMEM. These SOSV dilutions were then added to blood, tissue (calf liver) homogenate, or DMEM using the same proportions and methods that were used for collecting blood, tissues, urine or swab samples from bats. The relative SOSV TCID_50_/mL (fluid samples) or g (tissue samples) equivalents were interpolated from the relevant standard curve. The standard curve for swab samples was based on a calculation in which 20 μL of virus solution was absorbed by the swab applicator.

### Virus isolation

Virus isolation was performed as described previously [20] on all qRT-PCR positive oral and rectal swab, select tissue samples, as well as both positive urine and untested fecal samples that were collected opportunistically. Samples were placed into 500 μL DMEM/fungizone/penstrep (100 units/mL penicillin; 100 μg/mL streptomycin; 2.50 μg/mL amphotericin B; Life Technologies) with 2% fetal calf serum and flash frozen in liquid N2 until virus isolations were attempted. The entire eluate was used to inoculate Vero-E6 cells in 25 cm^2^ flasks for 1 h at 37 °C and 5% CO2. For fecal and colon/rectum samples, the initial supernatant was pre-treated with 5X antibiotic/fungizone additive, incubated for 1 hour at room temperature, and centrifuged prior to inoculation. Media was then replaced with DMEM containing 2% fetal calf serum, antibiotics and fungizone, and monitored for 14 days with a media change on days 1 and 7. All cultures were tested by immunofluorescent assays at 7- and 14-days post-tissue culture inoculation following published procedures [20] modified for the detection of SOSV using a polyclonal rabbit antibody raised against purified SOSV-N (GeneScript, Piscataway,New Jersey).

### Serology

Blood samples taken for serologic analysis were tested by indirect ELISA for the presence of IgG antibodies reactive to SOSV using a non-recombinant, infectious-based virus antigen lysate. SOSV antigen and uninfected control antigen lysates were prepared as previously described [21]. Briefly, Vero-E6 cells were inoculated with SOSV and harvested when immunofluorescence assay indicated at least 90% of the cells were infected. Viral antigen lysate was prepared from the culture by detergent basic buffer extraction of infected cells. Uninfected control antigen lysate was prepared in the exact same manner, but in the absence of SOSV. The cut-off value for seropositivity was determined by plotting the adjusted sum ODs of 53 SOSV-naïve bats (15 bats in this study prior to SOSV inoculation and 38 other captive-bred, SOSV-naïve ERBs) and then calculating a value greater than the mean ± 3 standard deviations. A bat was considered to have been infected with SOSV and seroconverted at an adjusted sum OD of ≥ 0.30 with a confidence level of greater than 99.7%.

### Histopathology

Representative sections from formalin-fixed tissues were prepared for histopathology and sections were routinely processed, embedded in paraffin, sectioned at 4–5 μm, mounted on glass slides, and stained with hematoxylin and eosin and were reviewed by two board-certified veterinary pathologists. To localize SOSV genomic RNA in tissues, we performed ISH using the RNAscope^®^ 2.5 HD Reagent Kit—RED (Advanced Cell Diagnostics, Newark, CA, USA) and followed the manufacturer’s directions to visualize the signal in formalin-fixed paraffin-embedded (FFPE) intestines and salivary gland of bats that were positive for SOSV virus by qRT-PCR, as previously described [22]. In situ hybridization was performed by using riboprobes that target multiple genes of SOSV virus obtained from the published genome [11]. The probes were validated on SOSV–positive culture cells and on SOSV–negative control tissues, including tissues from the 3 negative control bats. A riboprobe targeting the dapB gene (Advanced Cell Diagnostics) was used as a negative control probe. To test the RNA quality of the FFPE samples, a medium-expressing housekeeping gene (peptidylprolyl isomerase B, PPIB) probe was applied to each sample tested for SOSV (*R*. *aegyptiacus* NCBI Reference Sequence XM_016141088.1).

## Results

### Sosuga virus causes systemic infection in ERBs in the absence of overt clinical illness

**Fig 1:**
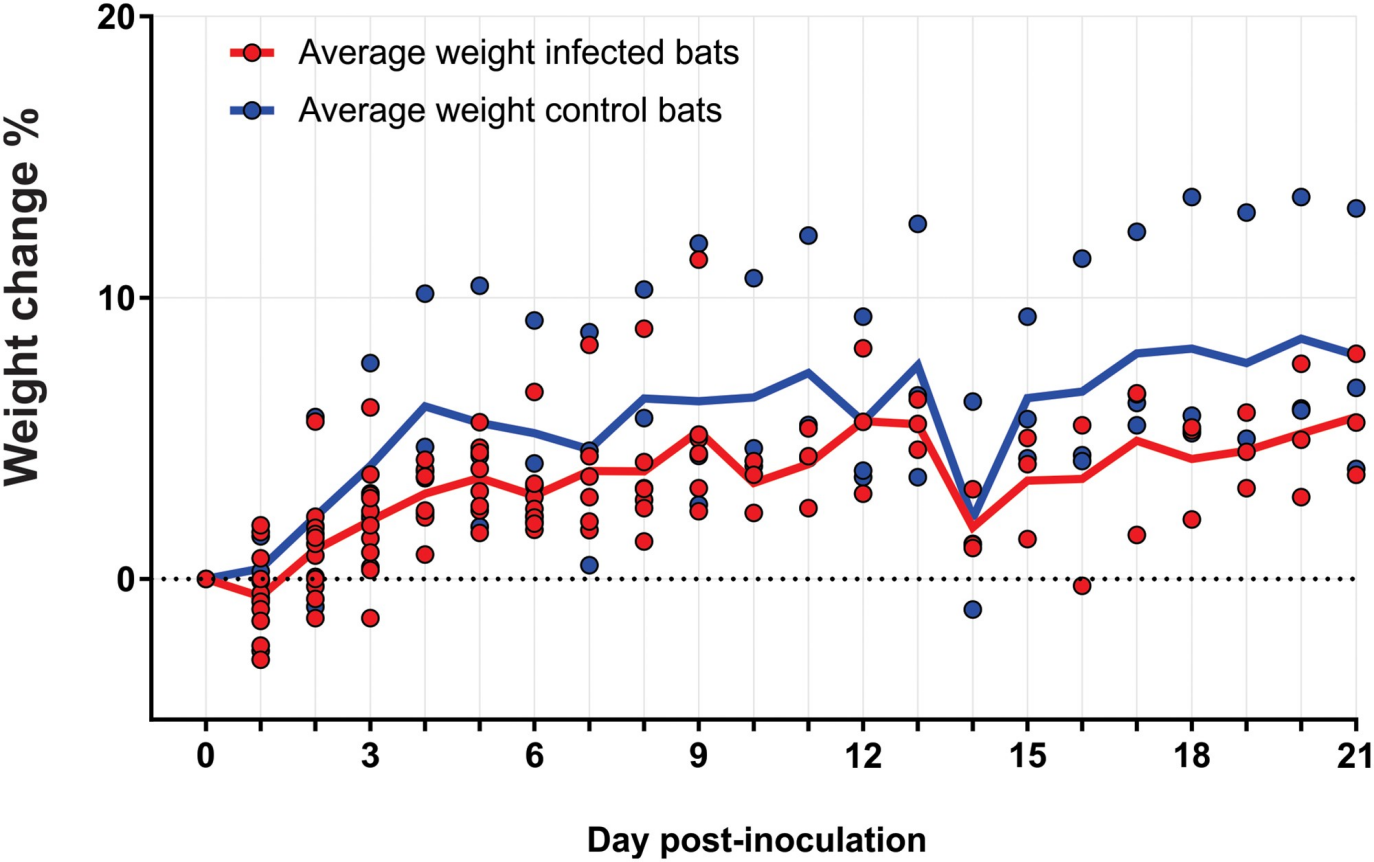
Percent weight change of inoculated and control bats. Percent weight change in grams comparison between experimental bats (n = 12; red) and negative control bats (n = 3; blue).

Following subcutaneous inoculation with SOSV, evidence of infection was detected in all of the experimentally inoculated bats (n = 12). However, no disease-related mortality or gross evidence of morbidity was observed in any of the bats throughout the experiment. There were no significant fluctuations in percent weight change between the experimentally infected and control bats (n = 3; Mann-Whitney test; p>0.1; Fig 1). Average body temperatures of infected bats were within normal range (39.5–40.5°C) and consistent with those observed in the control bats (40.1–40.2°C). No evidence of SOSV infection was detected in any of the control bats throughout the study by qRT-PCR for viral RNA or indirect ELISA for virus-specific IgG antibodies.

**Fig 2:**
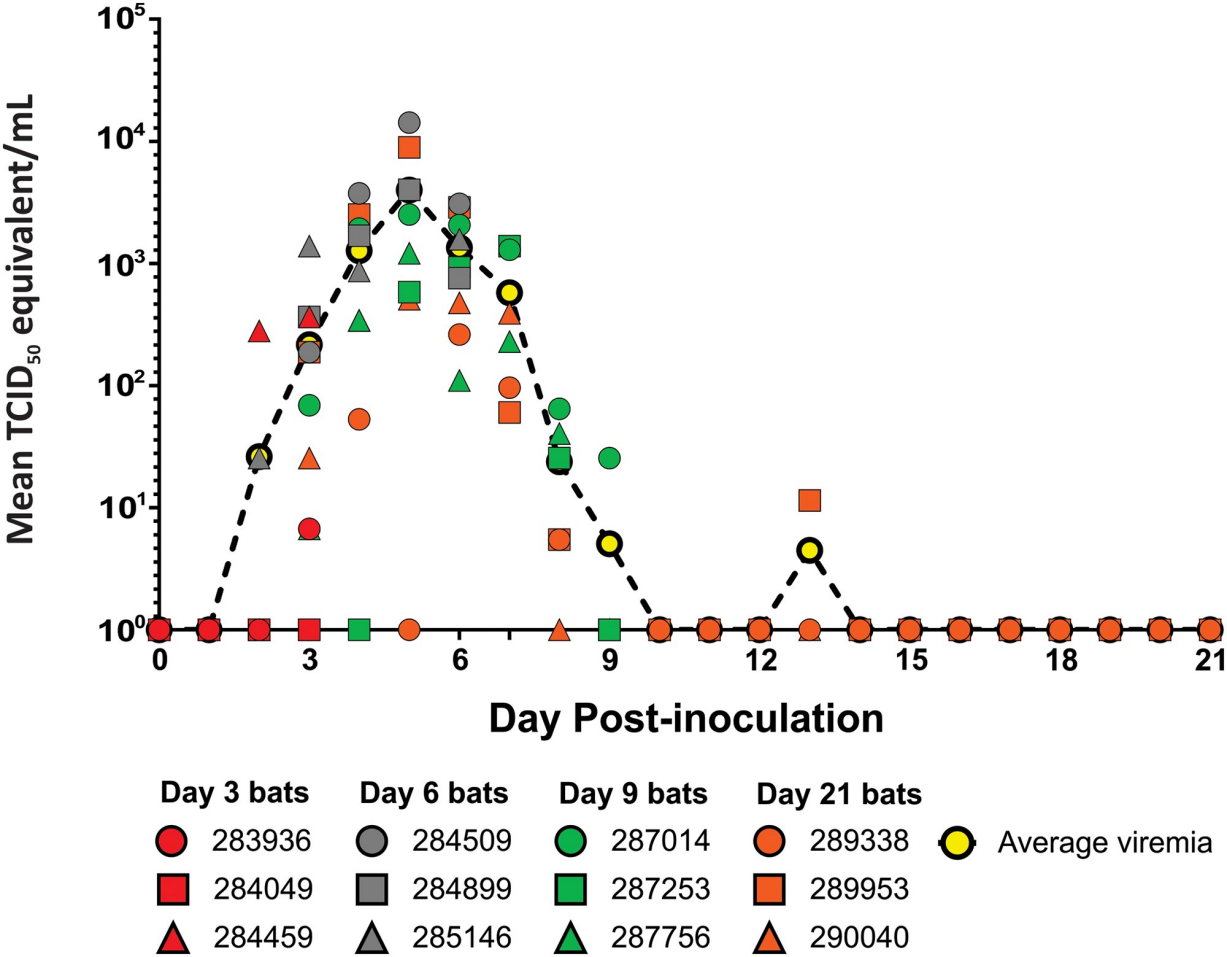
Sosuga virus RNA detected in blood of inoculated bats. Sosuga virus RNA levels in the blood (TCID_50_/mL equivalent) of individual bats that were sampled daily until their time of euthanasia. Individual bats are depicted by symbols (dot, square and triangle) and color-coded according to their day of euthanasia (red = day 3, gray = day 6, green = day 9, and orange = day 21). The total number of bats sampled according to days-post inoculation (DPI) were: 0–3 DPI = 12 bats, 4–6 DPI = 9 bats, 7–9 DPI = 6 bats, and 10–21 DPI = 3 bats. The mean viral RNA levels in the blood (TCID_50_/mL equivalent) for all bats according to DPI are shown as a dashed black line with yellow dots.

Viral infection reported in the experimental bats will hereafter be reported as TCID_50_ equivalents per gram of tissue or milliliter of fluid and represents the mean viral load in the tissue from the 3 bats sampled on that particular DPI. Viremia was detected in all but one bat (284049). Viremia peaked on 5 DPI with a mean high viral load of 3.99 x 10^3^ TCID_50_/mL (Fig 2). The average time span of detectable viremia for the experimental bats was 4 days, with a range of 2–7 days beginning on 2 DPI through 13 DPI.

**Fig 3:**
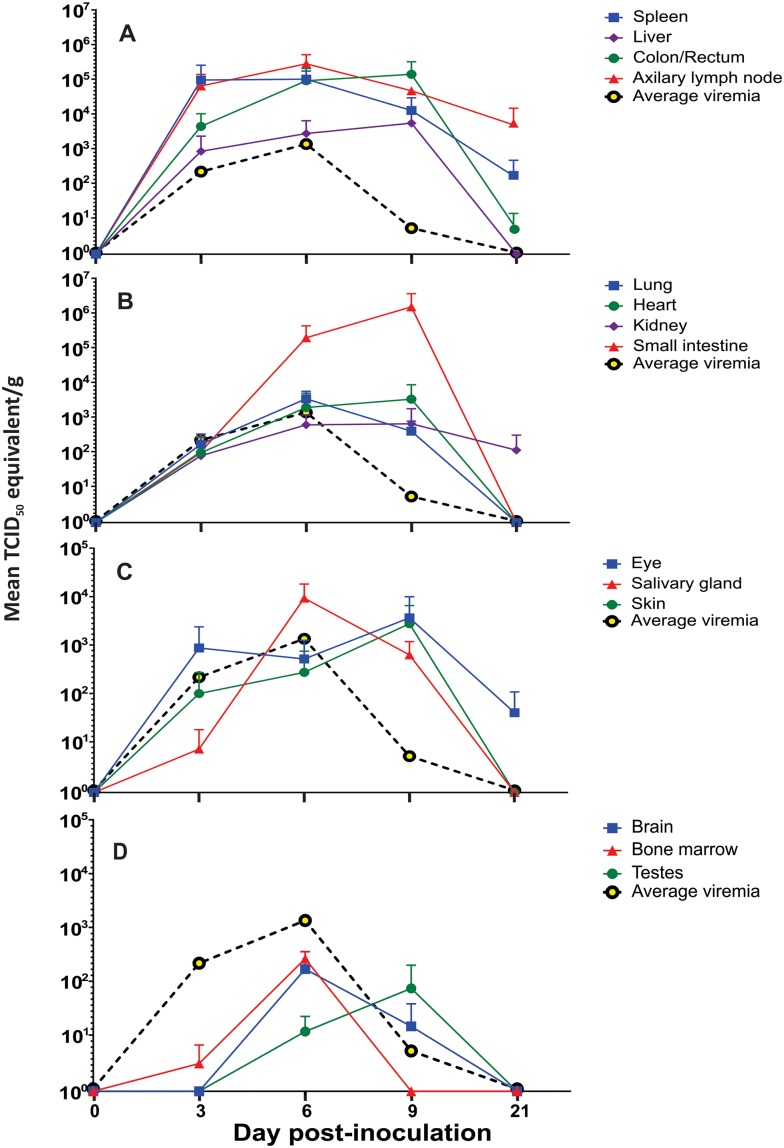
Sosuga virus RNA detected in tissues of inoculated bats. Mean Sosuga virus RNA levels (TCID_50_/g equivalent) in groups of three bats euthanized at 3, 6, 9- and 21-days post inoculation in (A) spleen, liver, colon/rectum, and axillary lymph node, (B) lung, heart, kidney, and small intestine, (C) eye, salivary gland, and skin at inoculation site and (D) testes, bone marrow and brain. Each symbol (point) represents the mean viral load detected in the corresponding tissue collected from the 3 bats euthanized on that day. For reference, the mean viral loads detected in blood from the same bats euthanized on that day are shown as a dashed black line with yellow dots.

Evidence of disseminated infection, as detected by qRT-PCR and relative to viremia, was found at some point in all but one tissue type sampled from inoculated bats (Table 1). As the bats were not perfused prior to necropsy, only tissues that exhibited viral loads higher than the corresponding blood specimen collected at euthanasia were considered positive. Viral loads in bone marrow did not exceed those loads found in the blood and therefore were not considered positive. Axillary lymph node and spleen had the highest viral loads early during the course of infection. Mean viral loads in the spleen peaked on 3 DPI (9.51 x 10^4^ TCID_50_/g) and in axillary lymph nodes on 6 DPI (2.78 x 10^5^ TCID_50_/g). Mean viral loads in colorectal tissue and liver also were detected as early as 3 DPI, but did not peak until 9 DPI (1.38 x 10^5^ TCID_50_/g and 5.40 x 10^3^ TCID_50_/g, respectively). Virus was cleared from the liver by 21 DPI, but remained detectable, albeit declining, in axillary lymph node (4.50 x 10^3^ TCID_50_/g), spleen (1.43 x 10^2^ TCID_50_/g), and colon/rectum (4.70 x 10^1^ TCID_50_/g) tissue (Fig 3A).

**Table 1:** Tissues qRT-PCR positive for Sosuga virus RNA.

Day	Bat	Sp	Li	ALN	CR	Ht	Ki	SI	Lu	Sk	SG	Ey	Ts	CNS	BM	Bl
3	283936	+++	--	+++++	++	--	++	--	++	--	--	--	--	--	--	+
	284049	+++	--	++	+++	--	--	--	--	--	--	--	--	--	--	--
	284459	+++++	+++	++++	++++	++	+	++	++	+++	+	+++	--	--	+	++
6	284509	+++++	+++	++++	++++	+++	++	+++++	+++	+++	++++	+	+	++	++	+++
	284899	+++++	++	+++++	+++++	++	+++	+++++	+++	--	+++	+++	+	++	++	++
	285146	++++	++	+++++	--	++	+	++++	+++	--	+++	++	--	--	++	+++
9	287014	++++	++++	++++	+++++	+++	+++	++++++	++	++++	++	++++	++	--	--	+
	287253	++	--	++++	+++	++	--	+++	+	--	+++	--	--	--	--	--
	287756	+++	+++	++++	++++	++	--	+++++	++	++++	+	--	--	+	--	--
21	289338	--	--	--	+	--	--	--	--	--	--	--	--	--	--	--
	289953	++	--	++++	--	--	--	--	--	--	--	++	--	--	--	--
	290040	--	--	+	--	--	++	--	--	--	--	--	--	--	--	--
Control	290195	--	--	--	--	--	--	--	--	--	--	--	--	--	--	--
	290494	--	--	--	--	--	--	--	--	--	--	--	--	--	--	--
	290631	--	--	--	--	--	--	--	--	--	--	--	--	--	--	--

The number of + symbols represents the viral load in TCID_50_/g (mL) equivalents derived from CT values generated by qRT-PCR: + = 10^1^ to ≤10^2^ TCID_50_/g (mL) eq., ++ = >10^2^ to ≤10^3^ TCID_50_/g (ml) eq., +++ = >10^3^ to ≤10^4^ TCID_50_/g (mL) eq., ++++ = >10^4^ to ≤10^5^ TCID_50_/g (mL) eq., ++++++ = >10^5^ to ≤10^6^ TCID_50_/g (mL) eq., -- = negative. Abbreviations are as follows: Sp = spleen, Li = liver, ALN = axillary lymph node, CR = colon and rectum, Ht = heart, Ki = kidney, SI = small intestine, Lu = lung, Sk = skin at inoculation site, SG = salivary gland, Ey = eye, Ts = testes, CNS = brain, BM = bone marrow, Bl = blood collected at euthanasia.

Mean viral loads in heart, lung, kidney and small intestines were also detectable at day 3 DPI, although at much lower levels (Fig 3B). Peak viral loads in lung were detected at 6 DPI (3.34 x 10^3^ TCID_50_/g) and in heart and kidney (3.27 x 10^3^ TCID_50_/g and 6.36 x 10^2^ TCID_50_/g, respectively) at 9 DPI. The highest mean viral load of all tissues sampled during the study occurred in the small intestine at 9 DPI (1.49 x 10^6^ TCID_50_/g). The virus was cleared in the lung, small intestine, and heart, but still detectable in the kidney at 21 DPI (1.10 x 10^2^ TCID_50_/g).

Mean viral loads for the salivary gland peaked at 6 DPI (9.29 x 10^3^ TCID_50_/g) and by 9 DPI for the skin at inoculation site and eyes (2.75 x 10^3^ TCID_50_/g and 3.35 x 10^3^ TCID_50_/g, respectively). Virus was cleared in the skin at the inoculation site and the salivary gland by 21 DPI, but the eyes remained positive although virus levels had declined (4.0 x 10^1^ TCID_50_/g; Fig 3C). Mean viral loads in brain tissue peaked at 6 DPI (1.69 x 10^2^ TCID_50_/g) and in the testes at 9 DPI (7.37 x 10^1^ TCID_50_/g), but notably, were lower than the peak viral loads detected in the blood at 6 DPI (Fig 3D). Viral loads in these tissues minimally surpassed the loads detected in the blood at 9 DPI (1.50 x 10^1^ TCID_50_/g and 7.34 x 10^1^ TCID_50_/g respectively). Infection in the brain and testes was not detectable at 21 DPI (Fig 3D).

**Fig 4:**
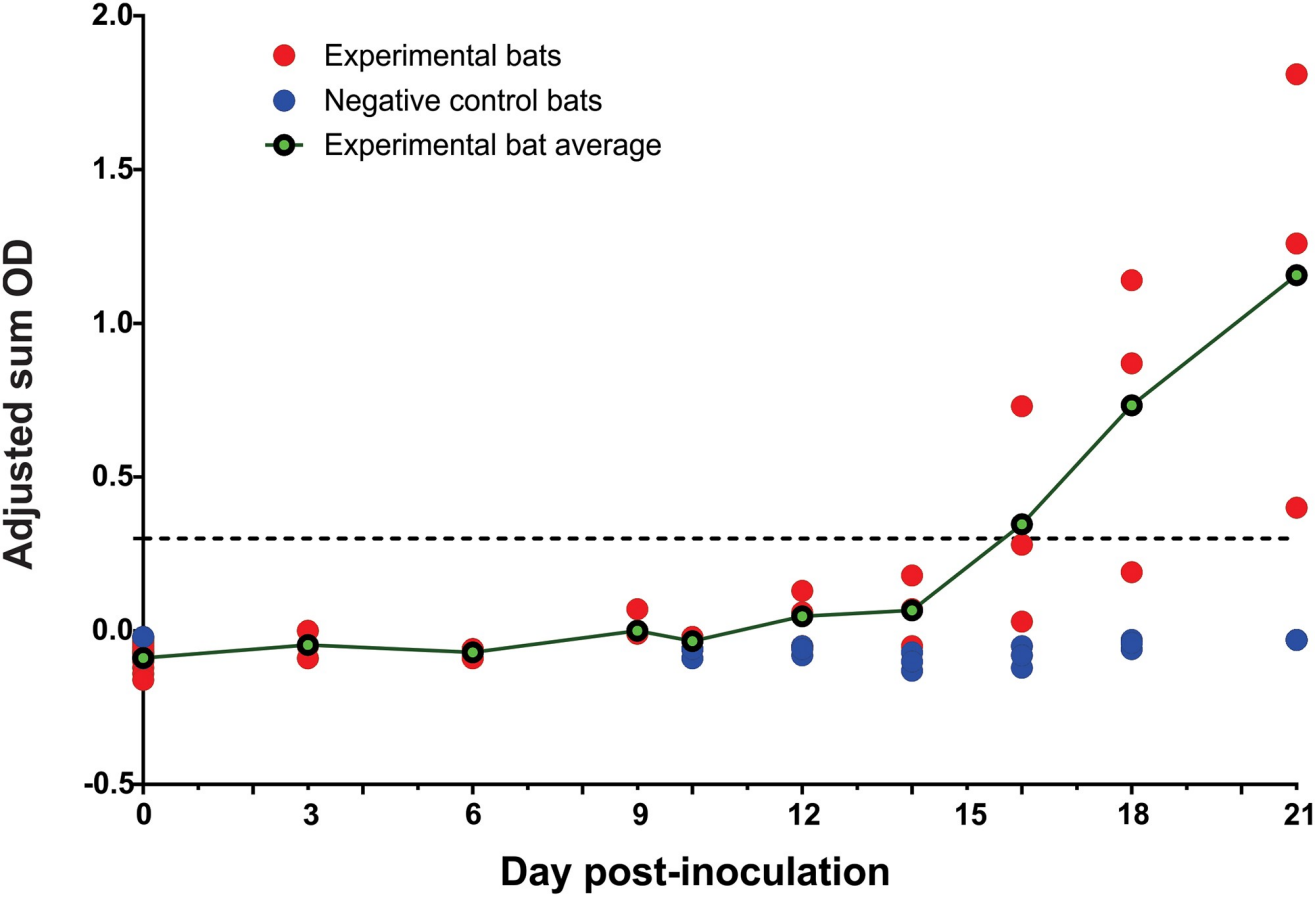
Sosuga virus antibody detected in inoculated bats. Serology results using an infectious based Sosuga virus antigen and anti-goat IgG. Sosuga virus IgG antibodies measured by indirect ELISA assay are shown as adjusted sum optical density (OD) values by day post-inoculation (DPI) for all infected bats (n = 12) at the time of euthanasia (0–9 DPI) and every other day beginning on 10 DPI for bats euthanized on 21 DPI. Results were considered positive (seroconversion) at a sum OD of ≥3.0.

Seroconversion was detected using an indirect ELISA with an adjusted sum OD positive cut-off value of 0.30. All three bats serially sacrificed at 21 DPI seroconverted to SOSV, with SOSV IgG antibody levels crossing the cut-off value of the assay between 16 and 21 DPI. As expected, all samples collected from the negative control bats tested uniformly negative for SOSV IgG antibodies (Fig 4).

### Mild tissue pathology is observed in the GI tract and salivary glands of Sosuga virus-infected ERBs

**Fig 5:**
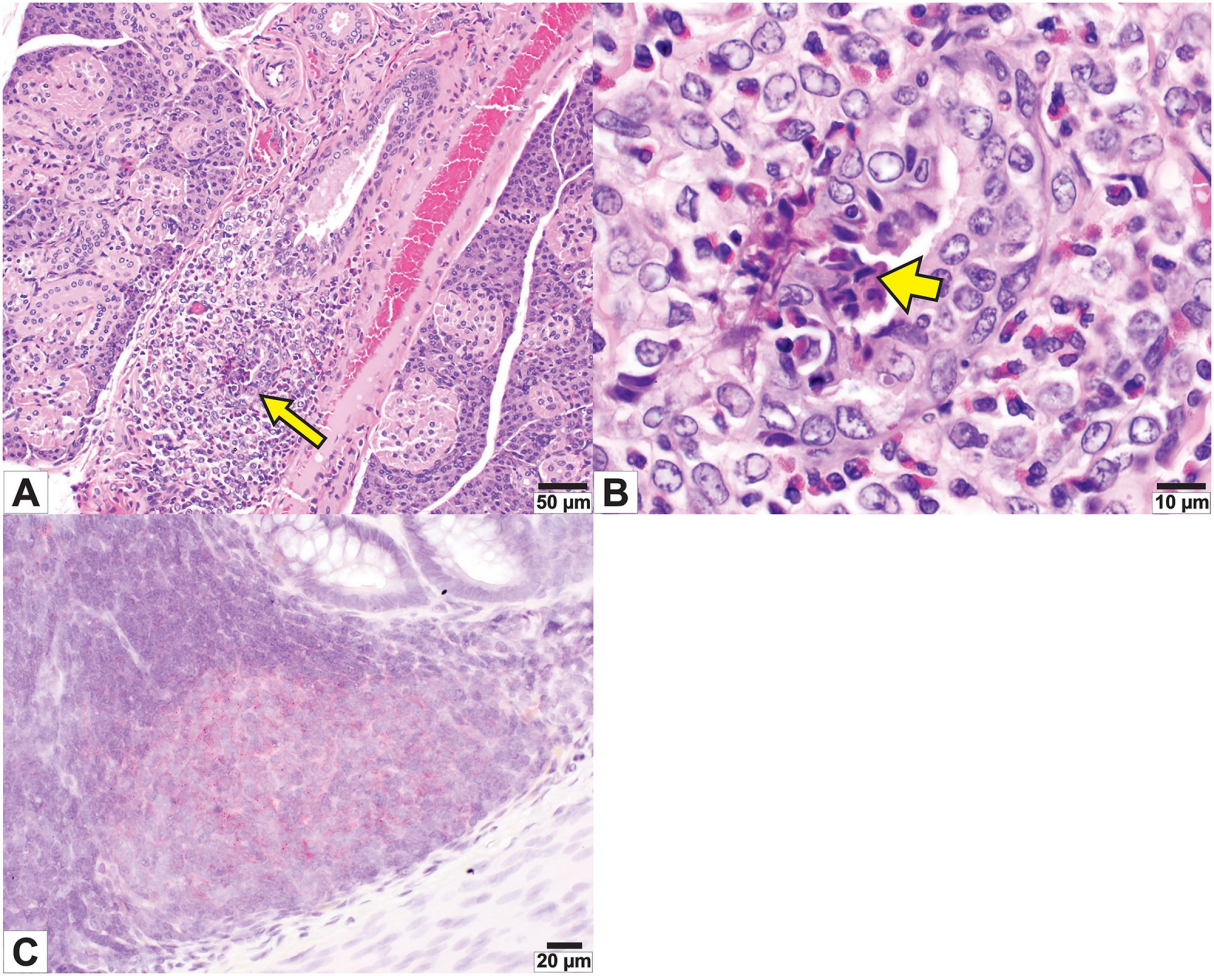
Mild pathologic effects of infection with Sosuga virus in the salivary gland and small intestine of inoculated bats. (A) Low magnification photomicrographs of the salivary gland from a 6-day post-inoculation (DPI) bat (284899) showing marked numbers of granulocytic inflammatory cells surrounding and infiltrating an intralobular duct (arrow). (B) Higher magnification photomicrographs of the same interlobular duct. Multiple ductular epithelial cells exhibit degeneration and few are necrotic, with hypereosinophilic cytoplasm and pyknotic nuclei (arrowhead). This bat was strongly qRT-PCR positive for SOSV in the salivary gland at necropsy (++++) and SOSV was detected in oral swabs at 5 and 6 DPI. (C) Positive hybridization signal for SOSV matrix protein gene (red chromogen) in the large intestinal gut-associated lymphoid tissue (GALT) from bat 289953 hematoxylin counterstain; original magnification 400x.

Histopathology revealed mild tissue changes in the small intestines (10 of 12, 83%) and salivary glands (6 of 12, 50%) of the SOSV-inoculated bats. One 6 DPI bat (284899) had severe inflammation within and surrounding the interlobular salivary gland ducts (Fig 5A and 5B). Further, this same 6 DPI bat had SOSV RNA in oral secretions at 5 DPI, the day prior to euthanasia. Additionally, at every timepoint assessed (3–21 DPI), the majority of SOSV-inoculated bats (83%) exhibited mild, multifocal, segmental expansion of the lamina propria at the small intestinal villus tips by homogenous, eosinophilic fluid, which elevated the epithelium and occasionally contained low numbers of inflammatory cells. There were mild degenerative changes in the overlying enterocytes, including multifocal loss of cell polarity and loss of the brush border. These changes were not observed in the three control bats. On in situ hybridization (ISH), positive hybridization signal for SOSV was localized to the large intestinal gut-associated lymphoid tissue (GALT; Fig 5C).

### Sosuga virus is detected in feces, urine, oral and rectal swabs of experimentally infected ERBs

**Fig 6:**
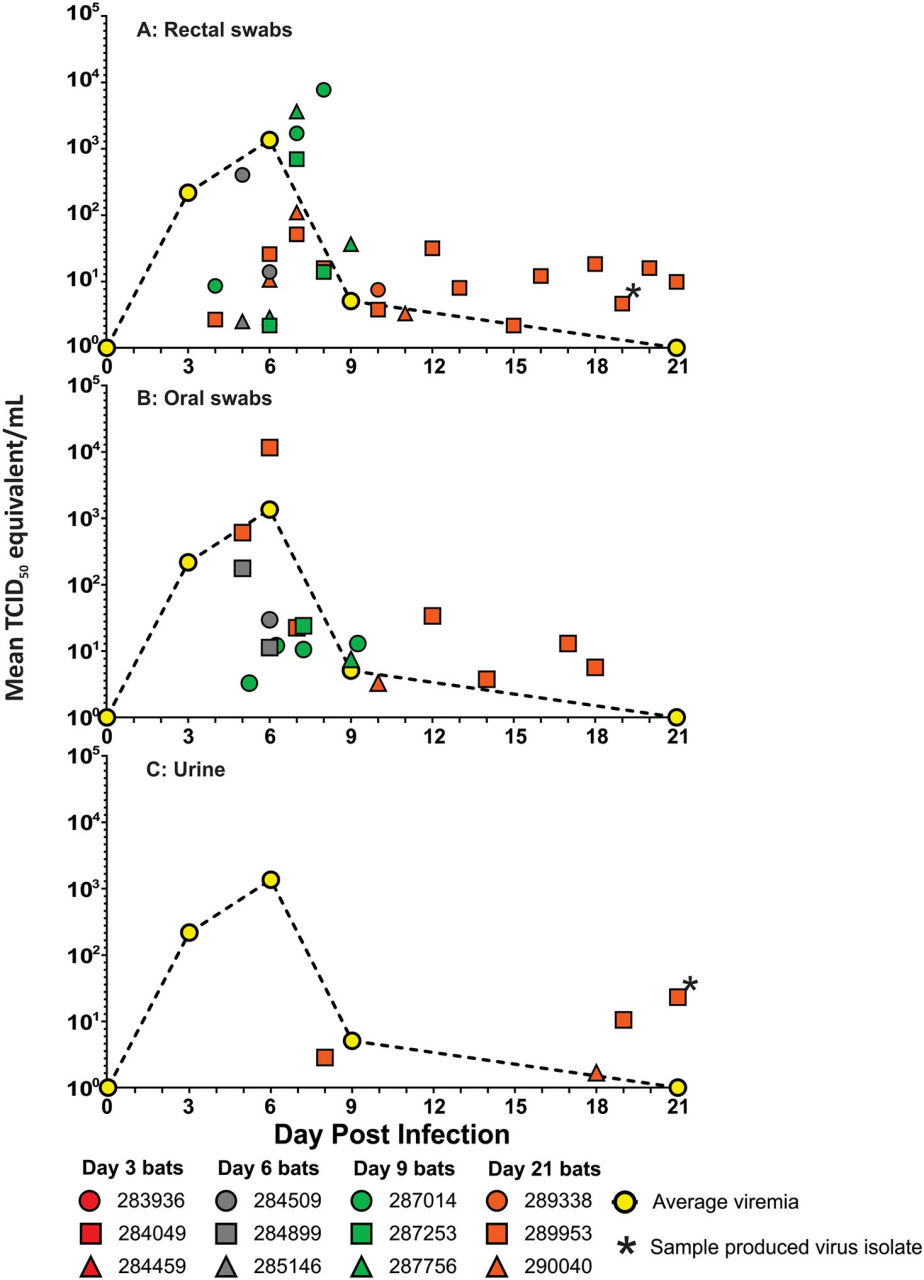
Sosuga virus RNA in urine and swabs (oral and rectal) from inoculated bats. Viral RNA levels (TCID_50_/mL equivalent) in (A) urine, (B) oral swabs and (C) rectal swabs by post-inoculation day. Infected bats are shown as one of three symbols (dot, square, or triangle) representing an individual bat sampled on a given day and then color coded for the day the bat was terminally sampled (red = day 3, gray = day 6, green = day 9, and orange = day 21). Swab viral levels (TCID_50_/mL equivalent) are equivalents of a SOSV stock tested (see Materials and methods), not the actual amount of virus in saliva or feces because the volume of absorbed oral secretion could not be precisely calculated. The rectal swab from which SOSV was isolated (panel A) is represented by an asterisk (*) by an orange square on day 19. Similarly, the SOSV isolate from a urine sample (panel C) is represented by an asterisk (*) by an orange square on day 21. For reference, the mean viral loads detected in blood from the bats euthanized on days 3, 6, 9, and 21 are shown as a dashed black line with yellow dots.

SOSV was first detected in rectal swabs (n = 30) beginning on 4 DPI and continuing through 21 DPI (Fig 6A). The highest viral load in a rectal swab was measured on 8 DPI (7.67 x 10^3^ TCID_50_/mL). SOSV-positive oral swabs (n = 17) were obtained beginning on 5 DPI with the highest viral load measured on 6 DPI (1.16 x 10^4^ TCID_50_/mL; Fig 6B). Virus was cleared from oral secretions by 18 DPI. SOSV RNA was also detected in urine samples (n = 4) collected opportunistically from 2 bats (Fig 6C), well after viremia had been cleared. The earliest positive urine sample was collected on 8 DPI (bat 289953) and viral loads continued to increase in urine samples collected from that bat until euthanasia at 21 DPI (2.31 x 10^1^ TCID_50_/mL). Virus isolations were performed on all qRT-PCR positive swabs, select qRT-PCR positive tissues including the spleen, kidney, colon/rectum, small intestine, as well as urine and fecal samples collected opportunistically throughout the study. SOSV isolates were obtained from feces (n = 3), urine (n = 1; Fig 6C), colorectal tissue (n = 2), rectal swab (n = 1; Fig 6A), and spleen (n = 1).

## Discussion

All 12 ERBs inoculated with the recombinant SOSV became infected. Viremia was detected in 11 of the 12 bats beginning on 2 DPI through 13 DPI. The one bat that was not viremic (284049) was euthanized on 3 DPI and would have most likely become viremic at some point in the study since 100% of infected bats were viremic at 6 DPI (9/9) and 7 DPI (6/6) and the same bat had SOSV qRT-PCR positive axillary lymph node, colon/rectum, and spleen at 3 DPI.

Similar to previous reports for ERBs infected with Marburg virus (MARV) [17, 20], we observed no changes in body weight, temperature, food consumption, or behavior to indicate significant clinical illness due to infection. Viral RNA was detected by qRT-PCR in visceral tissues of every inoculated ERB (Table 1). Spleen, axillary lymph node and colon/rectum were the most frequently qRT-PCR-positive tissues, but almost every tissue type sampled (n = 14) was SOSV qRT-PCR-positive at some point during this study (Table 1; viral loads in bone marrow did not exceed loads in blood). All tissues except the bone marrow (Fig 3A, 3B and 3C) showed viral loads higher than those detected in the blood, indicating that virus detection was due to viral replication in the tissue and not the result of viremia. Viral loads detected in bone marrow (peak = 8.08 x 10^2^ TCID_50_/mL) remained below the levels detected in the blood (peak = 8.08 x 10^2^ TCID_50_/mL) throughout the experiment. Viral loads in brain and testes did not surpass the peak levels detected in the blood until 9 DPI (Fig 3D), indicating very low-level viral replication in these tissues. The highest viral load for all tissues was seen in the small intestines (Fig 3B) at 9 DPI (1.49 x 10^6^ TCID_50_/g). This high viral load, coupled with viral RNA still being detected at 21 DPI in small intestine, kidney, and colon/rectum (Fig 3A and 3B) indicates these organ systems are potential routes of viral shedding and suggests mucus membrane contact with excretory/urogenital secretions from infected bats as a possible mechanism of SOSV transmission to humans or other animals. Viral loads also remained detectable, albeit declining, at 21 DPI in the liver, spleen, and eye (Fig 3A and 3C). Interestingly, immunoprivileged sites such as the eye have been associated with long-term viral persistence in humans infected with Ebola virus [23, 24]. No virus remained in testes, suggesting sex is not a major mechanism of bat-to-bat SOSV transmission.

Subclinical disease was observed in the infected bats and is consistent with observations in other competent hosts or natural reservoirs of zoonotic viruses [25–30]. Histopathology revealed mild lesions in the small intestines and salivary gland of several SOSV-inoculated bats (Fig 5A and 5B) during peak times of SOSV replication without outward signs of morbidity such as diarrhea or submandibular/cervical swelling, respectively. Lesions have been reported in experimental paramyxovirus infections of chiropterans, including inoculations of *Pteropus spp*. with HeV and NiV, causing vasculopathy in the gastrointestinal tract, kidney, spleen, placenta and lungs [9, 31, 32]. Vascular lesions were not observed in SOSV-infected ERBs in the current study. One 6 DPI bat had degeneration and inflammation of the interlobular salivary ductular epithelium, suggesting that this cell may be a target for SOSV entry and replication, but we were unable to localize SOSV RNA to this lesion using ISH. However, lack of hybridization signal on the positive control slide (PPIB) suggests poor RNA quality in the FFPE tissues for that animal (bat 284899). Accumulation of eosinophilic material in the lamina propria of the small intestinal villi of SOSV-infected bats was consistent with the tissue qRT-PCR results and the fecal shedding data, although the mechanism and significance of this fluid accumulation remains undetermined. Sosuga virus RNA was detected by ISH in the large intestinal GALT of bat 289953, but not in the small intestine.

Excretory and urogenital shedding of SOSV was demonstrated through numerous qRT-PCR-positive rectal swabs (n = 30; 2.17x10^0^–7.67 x 10^3^ TCID_50_/mL) and urine samples (n = 4; 2.83x10^0^–2.31x10^1^ TCID_50_/mL), in addition to virus isolation from fecal samples (n = 3), colorectal tissue (n = 12), urine (n = 1), and a rectal swab (n = 1). These routes of shedding could represent a primary mechanism of SOSV transmission to humans, but also between ERBs in a colony. As previously reported [18], ERBs observed in a large bat roost at Python Cave tend to urinate and defecate before and during initial flight, especially when disturbed, producing copious amounts of airborne, potentially infectious urine and fecal droplets. Moreover, juveniles in this particular colony, and possibly others, occupy roosting spots in holes on the floor of the cave where urine and feces are deposited regularly. Also, feces and urine are deposited nightly in large amounts in the vegetation nearby ERB roosts, and therefore this excretory-oral-mucosal route of transmission may also present a mechanism of spillover to humans and other animals visiting the roost site or occupying space nearby. Other paramyxoviruses like HeV and NiV are transmitted through mucus membrane contact with bodily fluids [33–35] or, as suggested by the qRT-PCR-positive lung tissue, through airborne droplets similar to the transmission of measles virus [14]. Transmission of SOSV through excretory and urogenital routes represents an even greater public health risk because direct contact with a bat is not required for transmission of the virus. Such transmission has been reported for other bat-borne viruses; for example, a Dutch tourist visiting Python Cave in Uganda became infected with MARV without being bitten by a bat or having any known pre-existing wounds exposed to bat excreta [36]. Similarly, the wildlife biologist infected with SOSV did not report being bitten by bats either [11], further suggestive of an excretory-mucosal route of infection for this virus.

In addition to SOSV shedding through excretory routes, virus also appears to be shed orally, as evidenced by the number of positive oral swabs (n = 17; 3.27x10^0^–1.16 x 10^4^ TCID_50_/mL) and salivary glands (n = 7; 7.11x10^0^–9.29 x 10^3^ TCID_50_/g). Moreover, viral RNA detected in the lungs (n = 8; 1.64x102–3.34 x 10^3^ TCID_50_/g) could also contribute to oral SOSV shedding. All together, these results suggest a potential oral bat-to-bat transmission route similar to that seen in ERBs infected with MARV [17]. ERBs live in tightly clustered colonies and have numerous squabbles over space and position [18, 37–39]. Males are known to subjugate females during mating through biting and perform muzzle licking during copulation [38]. Zoonotic disease transmission through aggressive encounters, including biting, have been reported for other animals [40, 41]. Viral shedding in oral secretions without biting may also play a role in the spread of SOSV. ERBs routinely test bite fruit to check for ripeness and drop fruit accidentally or spit out fruit pulp after mastication and removal of the juice [37], leaving potentially infectious material on the ground where it could be encountered by humans and other animals. Interestingly, at an outbreak of NiV, bats were seen drinking from date palm sap collection jars, some of which were also contained with bat excrement, thereby providing yet another potential mechanism of virus transmission [35].

An interesting outcome of this experiment was the disproportionately large contribution of one bat to the total number of SOSV-positive urine, and oral and rectal swabs. Bat 289953 was responsible for 43% of the SOSV-positive rectal swabs (13/30), 41% of the positive oral swabs (7/17) and 75% of the positive urine samples (3/4) as well as 3 virus isolates (2 fecal and 1 rectal swab). Furthermore, all positive swab and urine samples except one after 12 DPI was from bat 289953 (Fig 6A, 6B and 6C), suggesting that this bat could possibly be a ‘super-shedder’ [42]. Notably, captive bred, experimentally infected ERBs have also demonstrated this super-shedding capacity with MARV in accordance with the Pareto principle [20].

To fully establish that ERBs are indeed the *bona fide* SOSV natural reservoir, and not simply a competent intermediate host or part of a larger and more complex transmission cycle, additional field surveillance is needed to show that antibody and PCR positive bats can be found in ERB populations over time, and further that SOSV can be isolated directly from wild-caught ERBs. Such findings would help meet criteria for considering ERBs as a SOSV natural reservoir, currently defined as an ecological system in which an infectious agent survives indefinitely [43, 44]. Experimentation of longer duration is also needed to elucidate any persistence of infection in potential super-shedder bats, to conduct challenge trials in bats that have seroconverted, and to determine if factors such as age and co-infection with other ERB-borne viruses have effects on infection and viral shedding. Recently, large numbers of rubulaviruses were detected in ERBs in South Africa, but interestingly, not SOSV [45]. Nevertheless, the experimental SOSV infection study presented here, showing mild pathologic effects in infected ERBs along with many qRT-PCR and virus isolation positive tissues, as well as SOSV-positive urine, oral and rectal swabs, is consistent with natural reservoir expectations. Along with field data already collected, this study helps establish the ERB-SOSV relationship as a potential model experimental system for studying bat-borne paramyxovirus replication and shedding with documented human spillover potential.
